# A Comparative Study on the Use of Alprazolam and Melatonin for Sleep Disturbances in Hemodialysis Patients

**DOI:** 10.7759/cureus.11754

**Published:** 2020-11-28

**Authors:** Muhammad Sohaib Asghar, Muhammad Nadeem Ahsan, Rumael Jawed, Uzma Rasheed, Syed Anosh Ali Naqvi, Maira Hassan, Rabail Yaseen, Najia Mallick, Mahrukh Zehra, Muhammad Saleem

**Affiliations:** 1 Internal Medicine, Dow University of Health Sciences, Karachi, PAK; 2 Nephrology, Dow University of Health Sciences, Karachi, PAK; 3 Internal Medicine, Liaquat National Hospital, Karachi, PAK; 4 Internal Medicine, Dow International Medical College, Karachi, PAK; 5 Surgery, Dow University of Health Sciences, Karachi, PAK; 6 Psychology, Dow University of Health Sciences, Karachi, PAK

**Keywords:** chronic kidney disease, end-stage renal disease (esrd), hemodialysis, sleep-wake disorder, insomnia severity index, pittsburgh sleep quality index (psqi), daytime sleepiness, melatonin, alprazolam, benzodiazepine

## Abstract

Background and objectives

Sleep disorders are prevalent in end-stage renal disease (ESRD) involving the majority of patients undergoing hemodialysis. The main objective of treating sleep disorders in patients of ESRD is to correct subjective and objective sleep quality, decrease fatigue and daytime sleepiness, and enhance daytime functioning. Irrespective of the adverse effects reported, benzodiazepines are widely utilized among patients with sleep disorders in end-stage renal disease. Melatonin is a newer agent being studied for use in hemodialysis patients for improvement of sleep quality. The aim of our observational study is to witness the effectiveness of both benzodiazepine and exogenous melatonin as a treatment of sleep disorders in patients undergoing hemodialysis.

Materials and methods

We conducted a comparative, observational study in ESRD patients who are on hemodialysis. These patients were selected from attendees of the hemodialysis unit, nephrology department of a tertiary care hospital, including those who were on regular hemodialysis, thrice-weekly in frequency for at least once per year, and taking regular sleep medications for at least three months with frequently reported drug dosages of alprazolam 0.5 mg once daily or melatonin 3 mg once daily (before bedtime). The subjective sleep assessment was done by utilizing four scales, including the Pittsburgh Sleep Quality Index (PSQI), Epworth Sleepiness Scale (ESS), Insomnia Severity Index (ISI), and Stanford Sleepiness Scale (SSS).

Results

A total of 117 hemodialysis-dependent patients met the inclusion criteria, among whom 79 patients were using alprazolam while 38 were using melatonin for their disturbed sleep. The mean age of the study participants was 49.12 ± 12.75, comprising 72 males (61.53%) and 45 females (38.46%). The duration of the diagnosis of chronic kidney disease (CKD), duration of onset of hemodialysis, and estimated glomerular filtration rate (eGFR) was comparable in both groups. Both groups had similar laboratory markers except for higher hemoglobin in the melatonin group (p=0.028) and high parathyroid hormone (PTH) levels in the alprazolam group (p=0.001). PSQI scores were 8.76 ± 3.09 in the alprazolam group and 7.32 ± 2.65 in the melatonin group (p=0.015). In the sub-scores, there were no differences in sleep latency (p=0.481) and daytime dysfunction (p=0.662) while sleep efficiency (p=0.167) and subjective sleep quality (p=0.132) were not statistically significant. The significant differences were lower scores of sleep duration (p=0.040) and sleep disturbance (p=0.003) in the melatonin group. The ESS scores revealed no significant difference in either group (p=0.074). With respect to the ISI and SSS, higher scores were obtained in the alprazolam group. Overall, 89 study participants had reported poor sleep quality, out of which 81% were using alprazolam, and 65% were using melatonin (p=0.071). A total of 50 study participants exhibited excessive daytime sleepiness with 45% of them were using alprazolam and 36% were using melatonin. About 54% of the alprazolam using hemodialysis patients had moderate insomnia while 50% of the melatonin using patients had sub-threshold insomnia (p=0.062).

Conclusion

As melatonin use has shown better sleep quality and less insomnia severity as compared to alprazolam use in our study, it is postulated that the sleep-wake cycle should be commonly targeted by pharmacological therapy in ESRD.

## Introduction

Sleep disorders are prevalent in end-stage renal disease (ESRD), with 50-80% of patients undergoing hemodialysis and peritoneal dialysis complaining of disturbance in the sleep-wake cycle [1,2]. The etiology of sleep disturbances in sufferers of chronic kidney disease (CKD) is multifaceted, multiple conditions comprising of dialysis, medications, metabolic disturbances, malnutrition, exhaustion, muscle cramps, peripheral neuropathy, and emotional dilemmas are frequent in patients of chronic kidney disease [1-3]. Recurrent complaints extensively reported in patients of chronic kidney disease are insomnia, obstructive sleep apnea (OSA), restless leg syndrome (RLS), excessive daytime sleepiness, periodic movement of leg during sleep (PMLS), and frequent spontaneous awakening from sleep [1,2,4]. Prevalent sleep anomalies in patients with end-stage renal diseases are the delayed onset of sleep, persistent awakening, sleep fragmentation, and restlessness [2,4]. Insomnia is the most constant sleep abnormality reported by 50-75% of patients suffering from ESRD [5,6]. Increased frequency of insomnia in patients with CKD is due to excessive physical stress, chronic pain, increased age, restless leg syndrome, increased secretion of parathyroid hormone is also responsible for insomnia, and dialysis shift time plays a vital role in disturbance of sleep cycle prominent in patients with early morning appointments for dialysis as compared to patients scheduled for afternoon and night appointments [5]. Depression is a dominant mental health problem attributed to sleep disturbances with a 20-30% frequency in patients with CKD [7]. Pathophysiological mechanisms promoting sleep disorders among patients of ESRD are impaired sympatho-vagal tone with increased sympathetic stimulation and decreased parasympathetic activity leading to drooping of nocturnal blood pressure [5]. Patients suffering from ESRD or undergoing dialysis for it have decreased endogenous secretion of melatonin due to deterioration of kidney functions and a surge of melatonin is primarily decreased in patients scheduled for day shift of dialysis [8,9]. ESRD patients are reported to have a short distorted sleep cycle with a total sleep time of 260-360 minutes, an increased pattern of stage 1 and stage 2 of sleep with decreased slow-wave sleep and rapid eye movement (REM) sleep [5].

The main objective of treating sleep disorders in patients of ESRD is to correct subjective and objective sleep quality, decrease fatigue, daytime sleepiness, and enhance daytime functioning [2]. Treatment of sleep disturbances is categorized into two broad categories, non-pharmacological interventions comprising of sleep hygiene measures, relaxation therapy and biofeedback, stimulus control therapy, sleep restriction, and cognitive behavioral therapy beneficial for patients requiring long term treatment of insomnia along with persistent use of hypnotics and sedatives [2,5]. Pharmacological interventions used in the treatment of momentary insomnia comprise medications inducing sleep and improving daytime function in patients of ESRD [2,5,10]. Sedatives/hypnotics, sedating anti-depressants, anti-histamines, and exogenous administration of melatonin is used as a pharmacological treatment for insomnia [2,10]. The utilization of sedatives and hypnotics is moderate in patients undergoing dialysis with 8-10% frequency [10]. Benzodiazepines are commonly used and efficacious in treatment with multiple drugs for different phases of insomnia [2,11]. Benzodiazepines have demonstrated their effectiveness in curing insomnia of hemodialysis patients but adverse effects due to chronic use are also evident [2,11,12]. Irrespective of adverse effects been reported, benzodiazepines are widely utilized among patients with sleep disorders in ESRD due to their tendency of initiating and maintaining sleep [13].

Melatonin also denoted as “circadian hormone” is endogenously secreted from the pineal gland in a pulsating manner located in the hypothalamic suprachiasmatic nucleus, acting as a neuroendocrine inducer of the sleep-wake cycle [14]. The elevated surge of melatonin during the evening in normal conditions increases the evening tendency of sleep [15]. Melatonin is utilized to treat jet lag, insomnia, shift work, sleep-wake cycle disorders, and neurodevelopmental diseases. It is efficacious in induction and regulation of sleep, decreases sleep latency, increases total sleep duration, and improves sleep quality. Melatonin is opted for as an anxiolytic, sedative, analgesic, anti-hypertensive, and oncostatic [14]. Short-duration administration of exogenous melatonin in patients undergoing hemodialysis ameliorates sleep disorders thus improving the sleep-wake cycle by a decrease in sleep latency and boosting day functioning [9].

The use of alprazolam is quite prevalent in our study population. The aim of our observational study is to witness the effectiveness of both benzodiazepine and exogenous melatonin as a treatment of sleep disorders in patients undergoing hemodialysis.

## Materials and methods

We conducted a comparative, observational study in ESRD patients who are on hemodialysis. These patients were selected from attendees of the hemodialysis unit, nephrology department of a tertiary care hospital. The study included adult patients of both sexes, who were on regular hemodialysis, thrice-weekly in frequency for at least one year with no hospital admissions within the last two months, and taking regular sleep medications for at least three months. The most frequently reported drug dosages were alprazolam 0.5 mg once daily or melatonin 3 mg once daily (before bedtime). The study excluded patients with chronic liver disease, malignancy, heart failure, chronic obstructive pulmonary disease, obstructive sleep apnea, pregnancy, morbid obesity, endocrine dysfunction (hypothyroidism), and patients on antipsychotics, antidepressants, or hypnotics. Consents were obtained from the patients after explaining to them the objectives of the study. All patients were subjected to history taking, clinical examination, and neuropsychiatric examination during their visit to the hemodialysis unit. Laboratory investigations included hemoglobin, mean corpuscular volume, serum urea and creatinine, serum bicarbonate, serum calcium, serum phosphorus, parathyroid hormone levels, uric acid, and estimated glomerular filtration rate (eGFR).

Subjective sleep assessment was done by utilizing four scales. The Pittsburgh Sleep Quality Index (PSQI) denotes a sleep disturbance if the score is below 5. It consists of a 19-item scale used for the evaluation of subjective sleep quality. The 19 questions are combined into seven components; each gives a score of 0-3. All scores are added up to get a total score of 21, with higher scores signifying poor sleep quality. One component denotes the use of a sleep medication, hence was omitted in our study as all our participants were using regular sleep medications and would give a full score in this component. Hence, six components with a total score of 18 were evaluated in the participants. The PSQI has been a valid and reliable assessment supported by similar differences with polysomnography [15]. We used the scale in the local language with validity and reliability [16]. The Epworth Sleepiness Scale (ESS) is an eight-item scale of excessive daytime sleepiness. Each question has a Likert scale of 4 points having the possibility of sleep in different scenarios (with never = 0, and high chance = 3). All responses are summed up to give a total score of 24, with higher scores of greater than 10 signifying excessive daytime sleepiness [15]. The validated ESS scale in the local language was used [17]. The Insomnia Severity Index (ISI) is a seven-item scale that measures the severity of insomnia. It consists of a 5-point Likert scale to value each item as 0 = no problem and 4 = very severe problem, giving a total score of 28. The total score is interpreted as no insomnia (0-7), sub-threshold insomnia (8-14), moderate insomnia (15-21), and severe insomnia (22-28) [18]. The Stanford Sleepiness Scale (SSS) is another seven-point single item scale that measures subjective sleep quality throughout the day. It was used to assess the current sleepiness status of the participants while recording subjective responses.

## Results

A total of 117 hemodialysis-dependent patients met the inclusion criteria and were subsequently assessed for their sleep quality. Out of them, 79 patients were using alprazolam while 38 were using melatonin for their disturbed sleep. The mean age of the study participants was 49.12 ± 12.75, comprising 72 males (61.53%) and 45 females (38.46%). The duration of the diagnosis of CKD was 6.01 ± 3.88 years in patients using alprazolam and 5.55 ± 4.12 years in patients using melatonin (p=0.557). Similarly, the onset of hemodialysis was also similar in both study groups (p=0.741). A variety of laboratory markers were assessed that are known to cause sleep disruption in renal disease. Both the study groups had similar laboratory markers except for higher hemoglobin in the melatonin group (p=0.028) and high parathyroid hormone (PTH) levels in the alprazolam group (p=0.001). Estimated GFR (eGFR) was also similar in both groups as shown in Table 1.

**Table 1:** Comparison of basic and laboratory data between the groups (n=117). Data presented as Mean ± SD or frequency n(%). *indicates independent sample t-test used to compute the p-value. **indicates chi-square test used to compute the p-value. Abbreviations: CKD, chronic kidney disease; n, number of subjects; SD, standard deviation, PTH, parathyroid hormone; eGFR, estimated glomerular filtration rate.

Demographic data and Laboratory markers	Study groups		p-value
	Alprazolam (n=79)	Melatonin (n=38)	
Mean age (in years)	48.89 ± 13.62	49.57 ± 11.54	0.791*
Gender	Males: 45 (56.9%)	Males: 27 (71.0%)	0.142**
	Females: 34 (43.0%)	Females: 11 (28.9%)	
Duration of CKD diagnosis (in years)	6.01 ± 3.88	5.55 ± 4.12	0.557*
Onset of hemodialysis (in years)	2.54 ± 2.11	2.37 ± 3.40	0.741*
Hemoglobin (g/dL)	9.03 ± 1.46	9.78 ± 2.14	0.028*
Mean corpuscular volume (fL)	79.82 ± 5.21	81.21 ± 3.69	0.143*
Urea (mg/dL)	154.35 ± 62.49	167.15 ± 73.38	0.329*
Creatinine (mg/dL)	6.32 ± 1.46	6.63 ± 2.14	0.360*
Serum Bicarbonate (mEq/L)	17.95 ± 1.16	18.26 ± 1.29	0.195*
Serum Calcium (mg/dL)	7.51 ± 1.84	7.26 ± 1.03	0.437*
Serum Phosphorus (mg/dL)	4.37 ± 0.78	4.66 ± 1.14	0.110*
PTH (pg/mL)	196.36 ± 40.78	164.73 ± 29.78	0.001*
Uric acid (mg/dL)	7.05 ± 2.96	7.69 ± 3.47	0.303*
eGFR (ml/min/1.73m^2^)	15.12 ± 1.60	14.76 ± 2.11	0.308*

The patient’s assessment through PSQI scores revealed a total of 8.76 ± 3.09 in the alprazolam group and 7.32 ± 2.65 in the melatonin group (p=0.015). In the sub-scores, there were no differences between the two groups in sleep latency (p=0.481) and daytime dysfunction (p=0.662) while sleep efficiency (p=0.167) and subjective sleep quality (p=0.132) was slightly better in patients using melatonin but not statistically significant. The significant differences were lower scores of sleep duration (p=0.040) and sleep disturbance (p=0.003) in the melatonin group, hence showing overall improved sleep quality in comparison to the alprazolam group. The patient’s assessment through ESS scores revealed no significant difference (p=0.074) in the patients using melatonin (8.12 ± 4.43) as compared to the patients using alprazolam (9.88 ± 5.16). With respect to the severity of insomnia, higher scores were obtained in the alprazolam group which was statistically significant with the patients using melatonin (p=0.013). The SSS also revealed higher scores in the alprazolam group as compared to the melatonin group (p=0.003), as shown in Table 2.

**Table 2:** Comparison between both groups regarding PSQI, ESS, SSS, and ISI scores. All p-values calculated by independent sample t-test (*significant values of <0.05). Abbreviations: PSQI, Pittsburgh Sleep Quality Index; ESS, Epworth Sleepiness Scale; ISI, Insomnia Severity Index; SSS, Stanford Sleepiness Scale.

Sleep Scales		Alprazolam	Melatonin	p-value
		Means ± SD	Means ± SD	
PSQI	Subjective sleep quality	1.37 ± 0.84	1.14 ± 0.59	0.132
	Sleep latency	1.54 ± 0.99	1.41 ± 0.79	0.481
	Sleep duration	1.60 ± 0.75	1.31 ± 0.68	0.040*
	Sleep efficiency	0.96 ± 0.57	0.80 ± 0.61	0.167
	Sleep disturbance	1.81 ± 1.01	1.26 ± 0.64	0.003*
	Daytime dysfunction	1.47 ± 0.59	1.41 ± 0.87	0.662
	Total score (0-18)	8.76 ± 3.09	7.32 ± 2.65	0.015*
ESS		9.88 ± 5.16	8.12 ± 4.43	0.074
ISI		16.55 ± 7.12	13.21 ± 5.67	0.013*
SSS		2.53 ± 1.01	1.96 ± 0.78	0.003*

**Figure 1:**
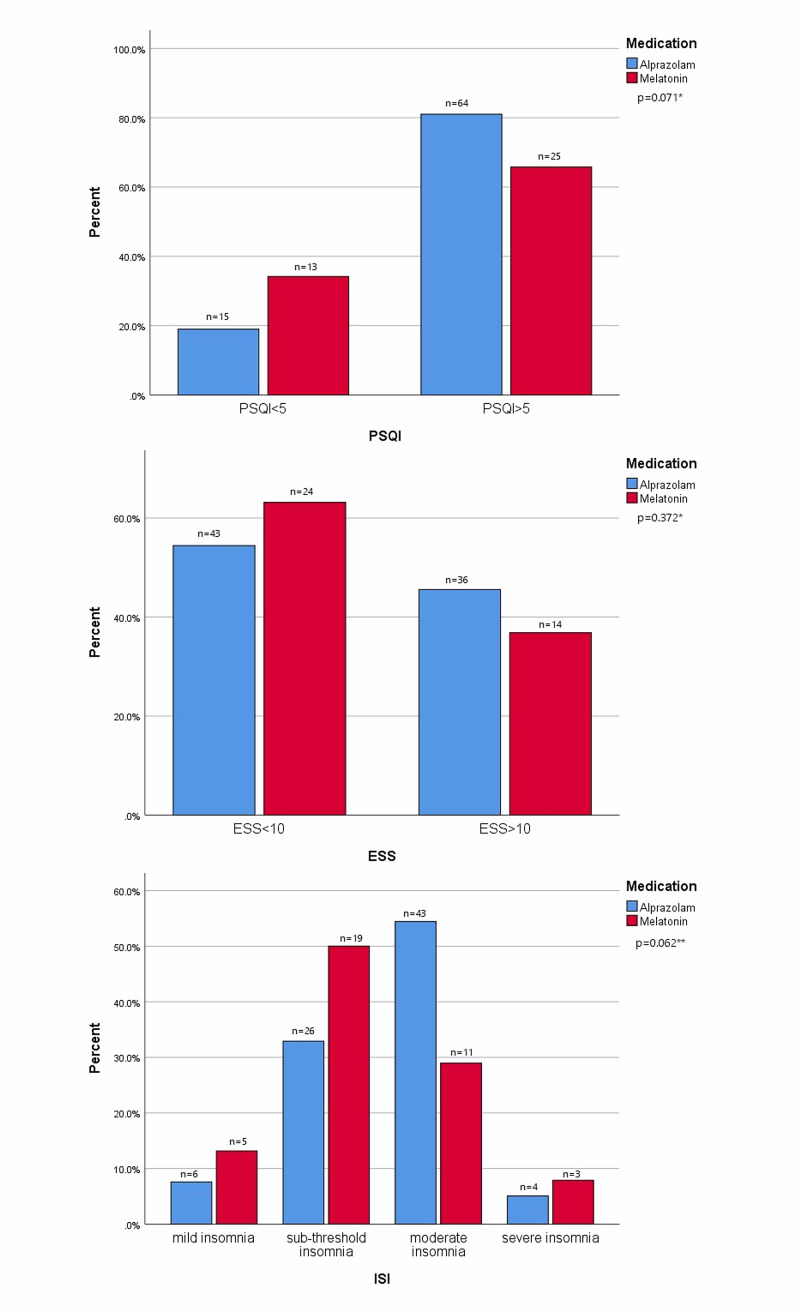
Comparison of frequency of descriptive sleep parameters among the study groups. *indicates chi-square test used to compute the p-value. ** indicates fisher's exact test to compute the p-value. Abbreviations: n, number of subjects; PSQI, Pittsburgh Sleep Quality Index; ESS, Epworth Sleepiness Scale; ISI, Insomnia Severity Index

Overall, 89 study participants reported poor sleep quality, out of which 81% were using alprazolam, and 65% were using melatonin (p=0.071). A total of 50 study participants exhibited excessive daytime sleepiness with no significant difference among the sleep medication been used (p=0.372). Around 45% of them were using alprazolam and 36% were using melatonin. With respect to the severity of insomnia, 54% of the alprazolam using hemodialysis patients had moderate insomnia while 50% of the melatonin using patients had sub-threshold insomnia (p=0.062). Better sleep quality and reduced insomnia severity in the melatonin group while indifferent daytime sleepiness in both groups is also shown in Figure 1.

## Discussion

The use of sleep medication in ESRD is quite prevalent, especially in hemodialysis patients, despite this many nephrologists are reluctant to use pharmacological means to address this issue. The use of sleep medication in ESRD patients has been reported to be as low as 3.6%, around 8-10% in a modest number of studies, while as high as 24% in one study [10]. The use of benzodiazepines is predominant in 8-26% of hemodialysis patients [19]. One such study associated it with fatigue and reported its use in 8% of the patients [20]. Another study conducted on 909 dialysis patients showed 17.7% (121 patients) were using benzodiazepines because of poor quality sleep [21]. A systemic review concluded the predominant use of benzodiazepines in hemodialysis patients as a sleep remedy [11]. Melatonin is a newer agent being studied for use in hemodialysis patients for improving sleep quality [8,9,19]. There is very limited data available regarding the use of medications for sleep disturbances in hemodialysis patients. In our study, the poor sleep quality reported by PSQI is 76.9%, which is similar to previously reported studies of hemodialysis patients [3]. Insomnia was reported higher in our study 90% using ISI, which is similar to reported before [22]. Excessive daytime sleepiness was found in 42.7% of hemodialysis patients using ESS, which was previously reported in 34% of dialysis patients using PSQI [22]. Another study reported insomnia in 25% and daytime sleepiness in 24% of hemodialysis patients, which was significantly lower compared to our results [23].

With regards to the subjective sleep parameters, we found a significant difference in insomnia with no significant difference in ESS scores in the patients using melatonin compared to the patients using alprazolam. The results of another study were similar to ours regarding the total PSQI scores being better in the melatonin group, as well as better sleep duration and lesser sleep disturbance [24]. The global PSQI score of 6.99 was near to our reported score of 7.32 in the melatonin-treated group. In comparison, their placebo group had a global PSQI score of 8.91 which was closer to our score of 8.76 in the alprazolam group. There were no differences between the two groups in sleep latency and daytime dysfunction while sleep efficiency and subjective sleep quality were slightly better in patients using melatonin but not statistically significant [24]. Another study used objective actigraphy to assess sleep quality in hemodialysis patients and their results were comparable with our study in improving sleep duration, sleep latency, and sleep disturbances [9]. In contrast to our results, a study conducted the long-term melatonin effects on sleep and quality of life in hemodialysis patients and found positive effects disappeared after six to 12 months [8]. Melatonin also significantly decreased depressive symptoms and anxiety during a period of three months according to a study conducted in post-operative breast tumor resection patients [25]. Melatonin treatment significantly improved depression scores as well as improved sleep quality [26]. Better sleep quality and less daytime sleepiness are associated with improved quality of life in hemodialysis patients [27].

Now coming to the benzodiazepines, their use has been linked with fatigue in hemodialysis patients [20]. While poor quality sleep has also been associated with decreased survival in hemodialysis patients, few studies have also shown the use of benzodiazepines increases the risk of death in hemodialysis patients [28-30]. The use of benzodiazepines is also associated with altered sleep quality during the first year of dialysis [21]. Despite being a reluctant option for sleep medication in hemodialysis patients, benzodiazepines are still a prevalent sleep medication being used in our study population. Regarding the scarcity of data, we found many studies comparing melatonin therapy with placebo, although no drug trial has been undertaken to compare the efficacy of different drugs. No study has rigorously examined the side effects of benzodiazepine use in dialysis patients. However, benzodiazepines were reported to be weakly associated with poor sleep quality [20-23,26] and reported mortality in a couple of studies [28,29].

There were limitations in our study including the use of subjective sleep assessments while no actigraphy or polysomnography (PSG) was performed for objective assessment. There was a limited sample size due to a discrepancy in the use of sleep medications among the study population. The exclusion criteria were strict in order to include only those patients undergoing regular hemodialysis and regular use of medications for optimum assessment of drug effectiveness. However, many patient factors other than CKD complications can contribute to sleep disturbances. The strength of the study was the inclusion of similar laboratory profile patients in both the groups, hence no external factors or bias in the reporting of sleep parameters.

## Conclusions

As melatonin use has shown better sleep quality and less insomnia severity as compared to alprazolam use in our study, it is postulated that the sleep-wake cycle should be commonly targeted by pharmacological therapy in ESRD. Although cognitive-behavioral therapy has also been slated to have a role in improving sleep disturbances in hemodialysis patients, drug dependence and renal clearance of prescribed medications are major factors to prevent pharmacological aid in hemodialysis patients. Conventionally, benzodiazepines are known for adverse effects and renal adjusted doses are not therapeutically beneficial to the patients either; melatonin is known for safer side effect profile and better therapeutic effectiveness. Further, large-scale randomized controlled trials are suggested to document the efficacy and tolerability of sleep medication in hemodialysis patients, as poor sleep is not only inducing anxiety and depression but also affecting the quality of life in chronic diseases, which is associated with early mortality.
